# Evaluation of pooling operations in convolutional architectures for drug-drug interaction extraction

**DOI:** 10.1186/s12859-018-2195-1

**Published:** 2018-06-13

**Authors:** Víctor Suárez-Paniagua, Isabel Segura-Bedmar

**Affiliations:** Computer Science Department, Carlos III University of Madrid, 28911 Leganés, Spain

**Keywords:** Deep learning, Convolutional neural network, Pooling, Attention model, Drug-drug interaction extraction

## Abstract

**Background:**

Deep Neural Networks (DNN), in particular, Convolutional Neural Networks (CNN), has recently achieved state-of-art results for the task of Drug-Drug Interaction (DDI) extraction. Most CNN architectures incorporate a pooling layer to reduce the dimensionality of the convolution layer output, preserving relevant features and removing irrelevant details. All the previous CNN based systems for DDI extraction used max-pooling layers.

**Results:**

In this paper, we evaluate the performance of various pooling methods (in particular max-pooling, average-pooling and attentive pooling), as well as their combination, for the task of DDI extraction. Our experiments show that max-pooling exhibits a higher performance in F1-score (64.56%) than attentive pooling (59.92%) and than average-pooling (58.35%).

**Conclusions:**

Max-pooling outperforms the others alternatives because is the only one which is invariant to the special pad tokens that are appending to the shorter sentences known as padding. Actually, the combination of max-pooling and attentive pooling does not improve the performance as compared with the single max-pooling technique.

## Background

Clinical trials are an essential phase of drug development process. They aim to test the safety and effectiveness of new drugs, however, these studies are not able to capture all the possible adverse drug reactions, and in particular, the drug-drug interactions (DDIs). A drug-drug interaction occurs when two or more drugs are taken at the same time and one of them alters the action or the effect of the others [1].

Doctors have at their disposal several databases and abundant pharmacovigilance literature that allow them to detect and prevent DDIs [2]. Every week, around 20,000 articles are published in PubMed (http://www.nlm.nih.gov/pubs/factsheets/medline.html). Pharmacology is one of the areas of biomedical research with a growing number of publications (300,000 articles per year) [3].

Natural Language Processing and Information Extraction (IE) techniques can help to lighten the workload of doctors by developing automatic systems capable to detect and extract relevant information from biomedical texts. The DDIExtraction shared tasks [4, 5] were two challenges organized for building systems and testing their performance in the extraction of DDIs from biomedical texts. Support Vector Machine (SVM) with linear and non-linear kernels were the most used systems and the techniques that obtained the state-of-the-art results with 77.5% for detection and 67% for classification [6], both in F1-score. A detailed description of the systems tested in the DDIExtraction-2013 challenge task can be found in [7]. SVM uses a large set of features predefined by text miners in order to get domain expert knowledge and generate a prediction. In the SVM systems for the DDI, the linguistic feature set was manually chosen consuming time and effort of the participants.

One of the main advantages of deep learning is its capacity to automatically infer the most informative feature set for a given task, such as text classification, named entity recognition or Relation Extraction (RE). The first deep learning model applied to RE was the Matrix-Vector Recursive Neural Network (MV-RNN) [8]. Concretely, this model outperformed the state-of-the-art techniques on the SemEval-2010 Task 8 dataset [9]. However, MV-RNN is not suitable for biomedical text because the parse trees generated by the Stanford Parser, which are used as the input, are often wrong due to the complexity of the sentence structures in this domain [10].

Recently, Convolutional Neural Networks (CNN) has received a big impact in many NLP tasks such as sentence classification [11], semantic clustering [12] and sentiment analysis [13]. This model uses filters to a matrix representation of the sentence in order to create a vector representation that learns the most relevant features for the task automatically. In the end, a classifier, like a softmax layer, is used to generate a prediction of a label to each vector. CNN was tested for an RE task in Zeng et al. [14] using the SemEval-2010 Task 8 dataset [9]. They used each word of the sentence and their relative position to the target entities transformed into a vector to create the input matrix for the CNN. They obtained a performance of 69.7% in F1-score and 82.7% by adding external lexical features before the classification.

Liu et al. [15] used the CNN model for the DDI corpus outperforming the rest of machine-learning techniques with an F1 of 69.75%. The architecture applied the convolutional function using the word embedding and the position embedding of each word of the DDI sentences in order to predict a possible relation between drugs. Recently, Suárez-Paniagua et al. [16] performed a detailed study of each CNN hyper-parameters for the DDI task. Furthermore, this work explored the random initialization of the word vectors, the use of different word embedding models and detail the results for each DDI type and for the two datasets of the DDI corpus, i.e. DDI-MedLine and DDI-DrugBank. The best performance was obtained with the random initialization of the input word vectors, the filter sizes (2, 4 and 6) and 10 dimensions for the position embeddings.

Liu et al. [17] extended their previous work [15] by performing the convolution operation on adjacent words in dependency parsing trees instead of on adjacent words in word sequences of candidate DDI instances [17]. Texts were tokenized by the NLTK toolkit [18] and the BLLIP parser [19] was used to obtain the constituent parsing trees for each candidate DDI instances. Then, these trees were transformed into dependency parsing trees by using the python package PyStanfordDependencies [20]. In the convolution layer, three convolution operations were performed. The first convolution operation has just been described above. The second convolution operation takes a word and its ancestor nodes in the dependency tree of the candidate DDI instance. Then the hyperbolic tangent function is applied on the concatenation of the word embeddings of the word and its ancestors. Similarly, the third convolution operation transforms a word, its father node and its sibling nodes in the dependency parsing tree. They combined their previous CNN and the Dependency CNN (DCNN), obtaining an F1 of 70.8%.

Likewise, Zhao et al. [21] also embed syntactic information into a CNN model for the DDI extraction task (SCNN). Concretely, the word embeddings are extended by including the position and part speech of each word. In the softmax layer, the convolutional features are combined with traditional features, the drug names, their surrounding words, the biomedical semantic types and the dependency types. This system achieved an F1 of 68.6%.

Most of the systems for relation extraction based on deep learning architectures take as input word embeddings. A word embedding model takes as input a large, unannotated text corpus (such as the last release of MedLine or a dump of the Wikipedia), constructs a vocabulary from the corpus and learns a vector representation for each word in the vocabulary. Based on distributional hypothesis [22], words which occur in similar contexts usually have similar meanings and will have similar vector representation. Therefore, these vectors (or word embeddings) are able to capture syntactic and semantic properties of words in the corpus. The work described in [23] presents, for the first time, an interesting CNN-based approach that combines five different word embedding models trained from five different corpora such as PubMed, PMC, MedLine and Wikipedia in a Multi-Channel Word Embedding (MCCNN). The combination of these models ensures a maximum coverage decreasing vocabulary gaps. The systems achieve an overall F1 of 70.2%.

To the best of our knowledge, there is only one work [24] that has applied a recurrent neural network with Long Short-Term Memory cells (LSTM) for DDI classification. This network is a sequential model that can keep information about the dependencies of the previous steps, which is very valuable in case of long sentences. In this work, they present three different LSTM based models for encoding the word embeddings pretrained on a PubMed corpus and position embeddings of the DDI sentences: B-LSTM computes the forward and backward states of the sentence, later a max-pooling operation is applied to the resulting matrix; AB-LSTM uses an attentive pooling layer on the LSTM output, and Joint AB-LSTM concatenates the outputs of these two previous systems before the classification. The experiments showed that attentive pooling method did not provide better results than those ones provided by a max-pooling layer. In addition, the combination of both operations provided a small improvement with respect to the use of a single max-pooling operation.

Several works have already been applied deep learning models to detect and classify DDIs, however, little attention has been paid to the pooling operations which are often incorporated into these architectures. Pooling is used to achieve more compact representations, preserving relevant features while removing irrelevant details. This layer can be performed in several ways, for example, calculating the average, taking the maximum, or as a linear combination of its inputs. All the previous CNN based systems for DDI extraction used max-pooling layers. Therefore, in this work, we aim to assess the effect of different pooling methods (such as max-pooling, average-pooling and attentive pooling) on the results of the task separately, but also their combination. Summing up, the main contribution of this paper is to give a comparative study of different pooling operations and their combination, on the performance of a CNN architecture for DDI classification.

Our hypothesis is that max-pooling for CNN is a very strong operation in which a lot of information of the generated filters is lost. For this reason, we explore and compare the effect of different pooling operations to try to combine all the filters extracted by the CNN in order to get all the information. In spite of LSTM models are suitable for sequence data as sentences, we think that the ability of the CNN for generating features from filters can generate a better representation of the sentences.

Table 1 summarizes the deep learning based systems for the task of DDI extraction. None of them has studied the effect of pooling operation on the performance of a CNN architecture for DDI classification task.

**Table 1:** Deep learning based systems results on the DDI corpus for the DDI classification task (best results in italic)

Systems	Approach	P	R	F1
Sahu and Anand [24]	Combined B-LSTM + AB-LSTM	73.41%	*69.66*%	*71.48*%
Liu et al. [17]	Combined CNN + DCNN	*78.24*%	64.66%	70.81%
Sahu and Anand [24]	B-LSTM	75.97%	65.57%	70.39%
Liu et al. [17]	MCCNN	75.99%	65.25%	70.21%
Liu et al. [17]	DCNN	77.21%	64.35%	70.19%
Liu et al. [15]	CNN with MEDLINE word embedding	75.72%	64.66%	69.75%
Zhao et al. [21]	Two-stage SCNN	72.5%	65.1%	68.6%
Zhao et al. [21]	One-stage SCNN	69.1%	65.1%	67%
Sahu and Anand [24]	AB-LSTM	67.85%	65.98%	66.9%
Suárez-Paniagua et al. [16]	CNN with random word embedding	69.86%	56.1%	62.23%

The paper is organized as follows: we describe in detail our architecture as well as the dataset used (see Method). Later, the experimental results are showed and a discussion section presents the possible reasons behind and the effects of each pooling operation on the task performance. Finally, we summarize our main conclusions and propose our future work.

## Method

### Dataset

**Fig. 1:**
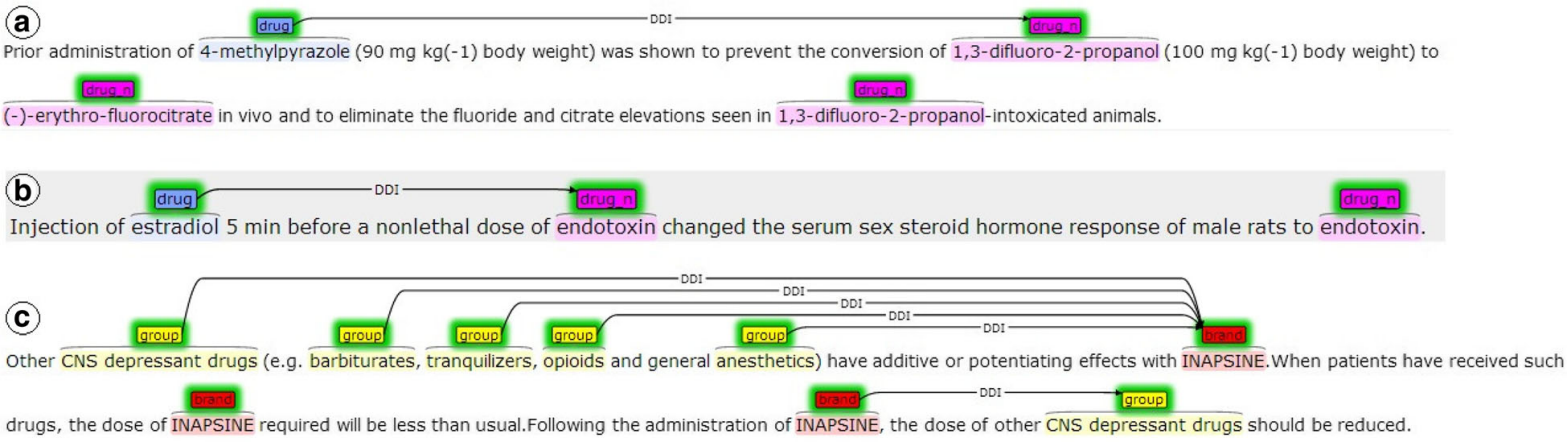
Some examples of sentences in the DDI corpus [7]. (**a**) describes a *mechanism*-type DDI between the drug (4-methylpyrazole) and the substance (1,3-difluoro-2-propranol). (**b**) describes an *effect*-type DDI between the drugs (estradiol) and (endotoxin) obtained in an experiment with animals. (**c**) describes several *effect*-type DDI with the drug (Inapsine) with five groups of drugs in the first sentence and (**c**) also describes an *advice*-type DDI of this drug with another group of drugs (CNS depressant drugs) in the third sentece

The DDI corpus [25] is considered a benchmark dataset for evaluating DDI extraction systems. It contains a total of 1025 documents, 233 Medline abstracts (DDI-MedLine) and 792 texts from the DrugBank database (DDI-DrugBank), which were manually annotated with 18,502 drugs and 5028 DDIs. In Fig. 1, the reader can find some examples of the DDI corpus in brat format (http://brat.nlplab.org/).

### CNN model

The proposed model is a CNN model based on [11], which was the first work to use this model for the sentence classification task. CNN is capable to transform each sentence into a vector in order to predict their class without the use of external resources. Concretely, the model applies filters to the input with different window size and create an output vector that describes the relevant part of the whole sentence. In the end, a classifier takes this vector as input to assign one of the labels.

**Fig. 2:**
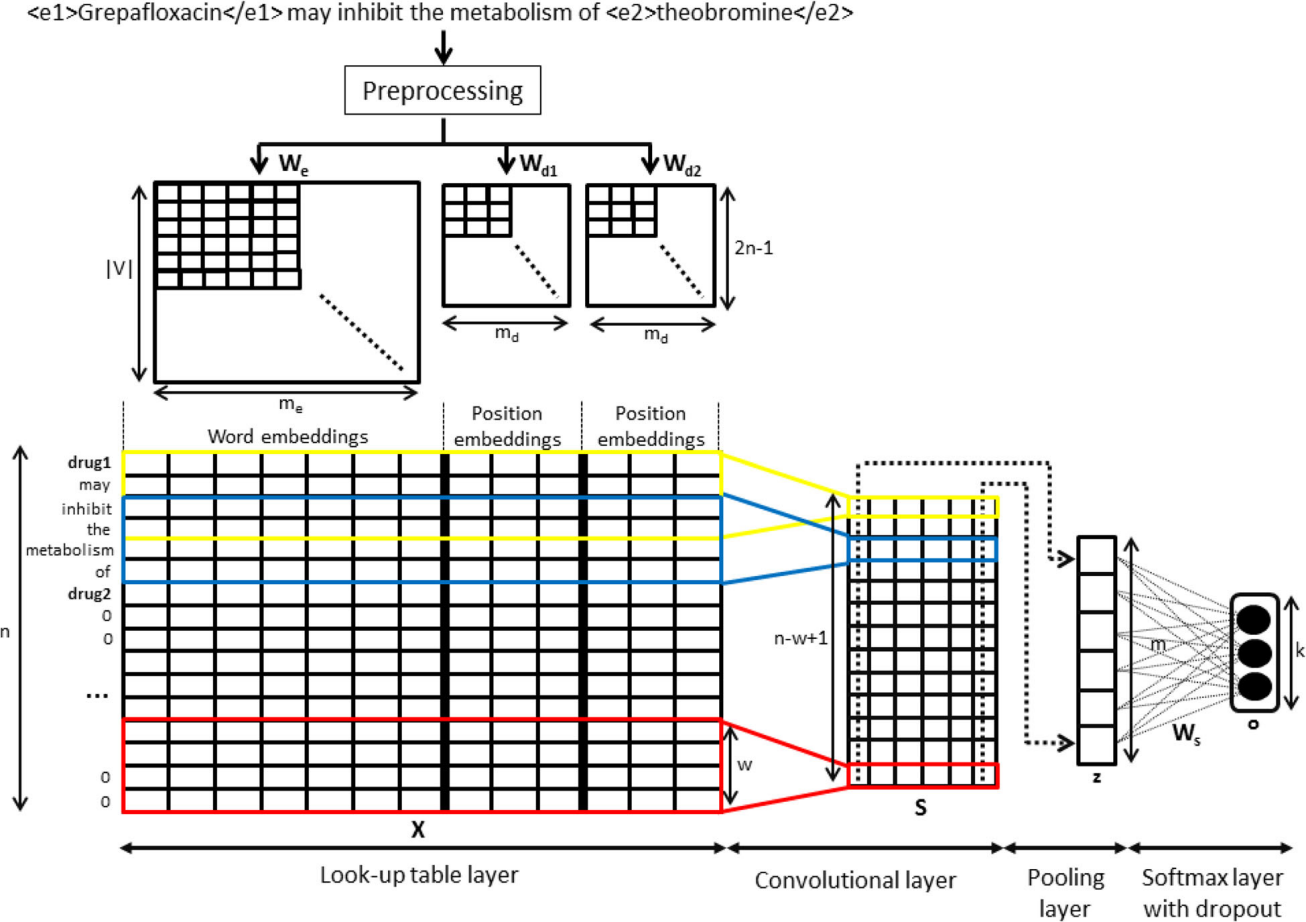
CNN model for DDIExtraction task

In this section, we present the CNN model for the classification of DDI sentences. Fig. 2 shows the entire architecture from taking a sentence with marked entities until the classification of each sentence into a DDI class.

### Pre-processing phase

A sentence with two drug names represents a relation instance, which is used as input to the CNN model. The training dataset contains a total of 27,792 relations instances. Only 4020 instances belong to the DDI category (that is, there is a total of 23,772 negatives instances). The test dataset contains a total of 5716 relations instances with only 979 positive instances and the remaining instances (4737) are negatives.

A relation instance can involve a discontinuous mention of a drug. For example, the following noun phrase *“ganglionic or peripheral adrenergic blocking drugs”* contains the drug name of *“peripheral adrenergic blocking drugs”* and the discontinuous mention of *“ganglionic adrenergic blocking drugs”*. There are some discontinuous drug mentions in the DDI corpus (only 47), which only produce a few numbers of instances (0.47%, 129 instances in the train set and 28 instances in the test set). In our CNN architecture, the representation of this kind of instances is not a trivial task and we decided to remove them.

Firstly, the DDI sentences were preprocessed as [11] using a tokenizer, converting them to lower-case and removing the special characters with regular expressions. In addition, the numbers are replaced by the label *NUM*. Moreover, we used the entity blinding, which guarantees the generalization of the model, replacing the target drug names that are interacting in each instance by the words “drug1” and “drug2”, and the remaining drug names by the word “drug0”. For example, the DDI sentence: *“Interaction on the antinociceptive effect between neurotensin and enkephalins or tuftsin”* should be transformed into the following relation instances:

1. *“Interaction on the antinociceptive effect between drug1 and drug2 or drug0”* for the relation *(neurotensin, enkephalins)*
2. *“Interaction on the antinociceptive effect between drug1 and drug0 or drug2”* for the relation (*neurotensin*, *tuftsin*)
3. *“Interaction on the antinociceptive effect between drug0 and drug1 or drug2”* for the relation (*enkephalins*, *tuftsin*)

However, we can claim that the last instance (*enkephalins*, *tuftsin*) cannot be a DDI because these drugs are conjuncts in the same coordinate structure. Therefore, we can rule out all of the instances that their drugs occur in a coordination. Similarly, we also discard all of the instances that their drugs occur in a hyponymous apposition [26]. An apposition is a noun phrase that follows another noun phrase and further describes or explains it. In a hyponymous apposition, the noun phrases are related by the relation of hyponymy. The following sentence shows an example of this kind of structure where the apposition is written in bold letters: *“Anticoagulants, such as heparin and warfarin, are often given prophylactically to prevent DVT”.* The relation instances (*Anticoagulants*-*heparin*), (*Anticoagulants*-*warparin*) and (*heparin*-*warfarin*) can be directly removed from the set of instances. Following the beneficial results of using a negative filtering preprocessing on DDI [6, 15, 27], we define a set of regular expressions that describe the structure of the most frequent coordinations and hyponymous appositions in the DDI corpus. Moreover, we also lighten the imbalance problem of the DDI corpus (almost 85% of instances are negatives).

These regular expressions achieve to automatically identify and rule out around 35% of negative instances (8409) from the training dataset and approximately 29% (1670) from the test dataset, whilst mistakenly filter out 150 and 32 positive instances from training and test datasets, respectively. At the end of this process, we got 19,233 relation instances (positives and negatives) to train the network and 4018 to test its performance.

### Word table layer

The pre-processed sentences are transformed into a matrix and are the inputs for the CNN model. These matrices should have the same length for being suitable for this architecture. We extended all the sentences adding an auxiliary token “*0*” until reaching the maximum length of a sentence in all training instances (denoted by *n*).

In addition, all the words in the sentences are represented by a vector taken from the word embedding matrix randomly initialized:${\mathrm{W}}_e\in {R}^{\left|V\right|\times {m}_e}$ where *V* is the vocabulary size and *m*_*e*_ is the word embedding dimension. After this process, we joined the word embedding vectors of all the words in the sentence in a matrix **x** = [*x*_1_, *x*_2_, …, *x*_*n*_] for each instance.

The relative position of each word with respect to the two interacting drugs are calculated as *i* − *p*_1_ and *i* − *p*_2_, where *p*_1_ and *p*_2_ are the positions of the two target drugs and *i* is the word position in the sentence. Furthermore, the range (−*n* + 1, *n* − 1) is regrouping to (1, 2*n* − 1) in order to avoid negative values. In the sentence shown in Fig. 2, the distances of the word “*metabolism*” to the two target drug entities “*Grepafloxacin*” and “*theobromine*” are 4 and − 2, respectively. Later, these relative distances are mapped into a real value vector using two position embedding ${\mathbf{W}}_{d1}\in {R}^{\left(2n-1\right)\times {m}_d}$ and ${\mathbf{W}}_{d2}\in {R}^{\left(2n-1\right)\times {m}_d}$. At the end of this process, we created a matrix $\mathbf{X}\in {R}^{n\times \left({m}_e+2{m}_d\right)}$ concatenating the word and the two position embeddings for each word in the sentence.

### Convolutional layer

The main operation of the convolutional neural network is performed in the convolutional layer. In this layer, we used a filter matrix $\mathbf{f}=\left[{f}_1,{f}_2,\dots, {f}_w\right]\in {R}^{w\times \left({m}_e+2{m}_d\right)}$ to a context window of size *w* for each instance represented by a matrix in order to generate features with a higher level of representation. We computed the score sequence **s** = [*s*_1_, *s*_2_, …, *s*_*n* − *w* + 1_] ∈ *R*^(*n* − *w* + 1) × 1^ for each filter applied to the sentence as

$$ {s}_i=g\left({\sum}_{j=1}^w{f}_j{x}_{i+j+1}^T+b\right) $$

where *g* is a non-linear function and *b* is a bias term. In Fig. 2, the total number of filters *m* with the same size of filter *w* is represented in a matrix **S** ∈ *R*^(*n* − *w* + 1) × *m*^. Nevertheless, in the case we use more than one size of filters, we would concatenate the resulting matrices of each filter size and fill each matrix with 0 for having the same size of the sentence length. Finally, we obtain a matrix $\mathbf{S}\in {R}^{\left({n}^{\ast }k\right)\times m}$ where *k* is the total number of different filter length.

### Pooling layer

The main goal of the pooling operation is to extract the most representative features of the sentence using a function that aggregates the output of each filter. It is a very important part of the architecture because it compacts the filtered information into a vector representation. This vector may capture the salient parts of the text and can be directly used as input of the classifier layer. For this reason, the selection of a correct pooling layer improves the final classification of the model. The aim of this article is to explore and select the best pooling operation for DDI and create a vector **z** = [*z*_1_,*z*_2_,…,*z*_m*k_], whose dimension is the total number of filters *m* by the number of different filter length *k* that represents the relation instance. In this study, we chose the following three different pooling layers that are applied to the output matrix of the convolutional layer.

### Max-pooling

The max function is the most common choice for the pooling layer in CNN architectures. This operation generates for each filter a single value as *z*_*f*_ =  *max* {**s**} =  *max* {*s*_1_, *s*_2_, …, *s*_*n*_}.

### Average-pooling

The average pooling is commonly used in image classification tasks but it is not very popular in NLP tasks. In this paper, we measure its performance for DDI extraction. In this case, the operation computes the average of each filter values: *z*_*f*_ = *mean*{**s**} = *mean*{*s*_1_, *s*_2_, …, *s*_*n*_}.

### Attentive pooling

The attentive pooling is a neural attention mechanism which focuses on the relevant words, capturing the important semantic information without using lexical resources or NLP tools. For this work, we follow the attentive pooling method proposed by [28] for the relation extraction task with LSTM. We have to adapt the attentive pooling model for a CNN architecture.

In the case of CNN, the operation uses a weight vector *w*^*T*^ ∈ *R*^*m* × 1^ which is multiplied by a filter normalization $M\in {R}^{\left({n}^{\ast }k\right)\times m}$. The resulting vector α ∈ *R*^*n* × 1^ determinates the relevant values of each word in the sentence for the classification.

$$ \mathrm{M}=\mathit{\tanh}\left(\mathbf{S}\right) $$

$$ \upalpha = Softmax\left(\mathbf{M}{w}^{\upalpha}\right) $$

Finally, the vector α is multiplied by the filter matrix **S** to reduce its dimensionality given by the relevancy of their words.

$$ {\mathbf{z}}^{\ast }=\mathit{\tanh}\left({\upalpha}^T\mathbf{S}\right) $$

### Softmax layer

In order to prevent overfitting, we performed a dropout before the classification. Firstly, we reduced the vector **z**_d_ randomly dropping some of the elements of **z** (z^*^ in the attentive pooling) with a probability *p* given by a Bernoulli distribution. Then, the prediction for each class is computed with the reduced vector in a softmax layer with weights **W**_*s*_ ∈ *R*^*m* × *k*^ as.

$$ \mathbf{o}={\mathbf{z}}_d{\mathbf{W}}_s+d $$

where *d* is a bias term; we have *k* = 5 in the dataset, corresponding to the classes *mechanism, effect*, *advice*, *int*, and non-DDI. The vector **z** is classified at test time by the softmax layer without a dropout for new examples.

### Learning

The CNN parameter set trained in the training phase are Θ = (**W**_*e*_, **W**_*d*1_, **W**_*d*2_, **W**_*s*_, **F**_*m*_), where **F**_*m*_ are all of the *m* filters **f**. We used the conditional probability of a relation *r* obtained by the softmax operation as

$$ \mathrm{p}\left(r\left|\boldsymbol{x},\Theta \right.\right)=\frac{\exp \left({\mathrm{o}}_r\right)}{\sum_{l=1}^k\exp \left({\boldsymbol{o}}_l\right)} $$

to minimize the log-likelihood function for all instances (**x**_*i*_, y_*i*_) in the training set *T* as

$$ J\left(\Theta \right)={\sum}_{i=1}^T\log\;p\left({y}_i\left|{x}_i,\Theta \right.\right) $$

In the training phase, we used the stochastic gradient descent to reduce the error of the objective function over shuffled mini-batches and the Adam update rule [29] to learn the parameters. In addition, we added *l*_2_-regularization in order to prevent over-fitting for the weights of the softmax layer **W**_s_.

## Results

Our previous work [16] aimed to provide an in-depth study of the influence of the CNN hyper-parameters. In this paper, we apply the same CNN architecture, but also explore the effect of new pooling operations (average and attentive). We use the same hyper-parameters values provided by [16] because they were obtained using the same dataset and architecture.

- Maximal length *n* = 128.
- Word embedding initialization W_*e*_*=random*.
- Word embedding size m_e_=300.
- Position embedding initialization **W**_d1_, **W**_d2_ = *random*.
- Position embedding size m_d_=5.
- Filters for each window size *m* = 200.
- Filter size *w* = (2,4,6).
- Mini-batch size =50.
- *l*_2_-regularization =3.
- Dropout rate *p* = 50%.
- Rectified Linear Unit (ReLU) as the non-linear function *g*.

In this previous work, it was needed to randomly select 2748 instances (10%) from the training dataset as our validation set. Due to the fact that we already know the best values for the hyper-parameters, we do not need to validate our model. Therefore, we can use the entire training set to train our CNN model. The results for all of the categories in the classification were measured using the Precision (P), Recall (R) and F1-score (F1).

Tables 2 and 3 show the performance of the max-pooling layer without and with negative instance filtering, respectively. Tables 4 and 5 show the results using the average-pooling and the attentive pooling, respectively, and Table 6 shows the performance obtained using the combination of two parallel CNN models with the max-pooling layer and the attentive pooling.

**Table 2:** Results obtained for max-pooling CNN on the test dataset without negative filtering

Classes	P	R	F1
Advise	79.33%	64.25%	71.00%
Effect	68.90%	54.17%	60.65%
Int	81.08%	31.25%	45.11%
Mechanism	58.29%	70.57%	63.84%
Overall	67.13%	59.22%	62.93%

**Table 3:** Results obtained for max-pooling CNN on the test dataset with negative filtering

Classes	P	R	F1
Advise	80.36%	61.09%	69.41%
Effect	62.06%	64.15%	63.09%
Int	62.32%	44.79%	52.12%
Mechanism	67.24%	66.11%	66.67%
Overall	67.19%	62.14%	64.56%

**Table 4:** Results obtained for average-pooling CNN on the test dataset with negative filtering

Classes	P	R	F1
Advise	66.99%	63.35%	65.12%
Effect	58.14%	63.03%	60.48%
Int	66.67%	31.25%	42.55%
Mechanism	61.90%	47.99%	54.06%
Overall	61.70%	55.35%	58.35%

**Table 5:** Results obtained for attentive pooling CNN on the test dataset with negative filtering

Classes	P	R	F1
Advise	78.74%	61.99%	69.37%
Effect	58.29%	57.14%	57.71%
Int	79.07%	35.42%	48.92%
Mechanism	60.75%	54.03%	57.19%
Overall	64.42%	55.14%	59.42%

**Table 6:** Results obtained for the combination of max-pooling and attentive pooling CNN on the test dataset corpus with negative filtering

Classes	P	R	F1
Advise	79.23%	65.61%	71.78%
Effect	65.28%	61.62%	63.40%
Int	80.49%	34.38%	48.18%
Mechanism	69.23%	60.40%	64.52%
Overall	70.40%	59.47%	64.47%

## Discussion

In this section, we evaluate the different pooling operations, their combination and also study the effect of negative filtering on the performance.

### Max-pooling

The results using the max-pooling CNN without negative filtering are very similar to [1818] because we use exactly the same configuration (see Table 2). However, we now obtain a higher F1 (62.93%) because we use the entire training dataset.

Table 3 shows the results with the negative filtering, which increases the overall performance in F1 over the previous experiment (+ 1.63%). Negative filtering also improves the performance of three of the four DDI classes, except for the advise type, where F1 is slightly lower and not significant. In the remaining experiments, we decided to apply negative filtering.

### Average-pooling

As can be seen in Table 4, all performance measures present a drastic decrease, especially in recall, when we used average pooling layer instead of a max-pooling one. A possible reason may be the padding operation. In shorter sentences, the average-pooling may disrupt the representation caused by the appending of the special pad tokens.

### Attentive pooling

Similarly to the average-pooling results, the attentive pooling layer (see Table 5) does not achieve better results than those obtained with max-pooling. Even so, the results are better than those ones using average-pooling results. In this case, the negative effect of padding on the results may be much lower than in the average-pooling because the weight of PAD tokens is possibly much smaller than the rest of the tokens.

### Pooling combination

We train two separated models using two different pooling operations (in particular, max-pooling and attentive) and concatenate the two pooling vectors into a single vector, which is the same method of [24] but applied to the CNN output. The resulting vector of this operation is the input of the softmax layer for the final classification of each instance. Table 6 shows the results of this combination. Neither the combination of the max and attentive pooling operations overcomes the use of a single max-pooling layer.

## Conclusions

In this work, we compare three different pooling operations for the task of DDI extraction. Our experiments show that the best operation is max-pooling. Attentive and average pooling operations provide worse results possibly caused by the negative effect of special pad tokens that are appending to the shorter sentences. In future work, we plan to ignore the pad tokens in the implementation of our attentive and average pooling operations. Contrary to other previous works we are using the random initialization of the word embeddings, thus we plan to explore pretrained word embedding over some biomedical resources to the attention model. We also plan to use a multi-channel word embedding by integrating several word embeddings models.

## Abbreviations

AB-LSTM: Attention pooling B-LSTM
B-LSTM: Bidirectional long short term memory network
CNN: Convolutional Neural Network
DCNN: Dependency CNN
DDI: Drug-Drug Interaction
DNN: Deep Neural Network
F1: F1-score
IE: Information extraction
MCCNN: Multi-channel CNN
MV-RNN: Matrix-Vector Recursive Neural Network
NLP: Natural Language Processing
P: Precision
R: Recall
RE: Relation Extraction
ReLU: Rectified Linear Unit
SCNN: Syntax CNN
SVM: Support Vector Machine
